# Inter-rater reliability of stress signatures in exfoliated primary dentition - Improving scientific rigor and reproducibility in histological data collection

**DOI:** 10.1371/journal.pone.0318700

**Published:** 2025-03-19

**Authors:** Simone A. M. Lemmers, Mona Le Luyer, Samantha J. Stoll, Alison G. Hoffnagle, Rebecca J. Ferrell, Julia A. Gamble, Debbie Guatelli-Steinberg, Kaita N. Gurian, Kate McGrath, Mackie C. O’Hara, Andrew D. A. C. Smith, Erin C. Dunn

**Affiliations:** 1 Center for Genomic Medicine, Massachusetts General Hospital, Boston, Massachusetts, United States of America; 2 Department of Psychiatry, Harvard Medical School, Boston, Massachusetts, United States of America; 3 Elettra Sincrotrone Trieste S.C.p.A., Basovizza, Trieste, Italy; 4 National Science Foundation, Alexandria, Virginia, United States of America; 5 Department of Anthropology, University of Manitoba, Winnipeg, Manitoba, Canada; 6 Department of Anthropology, The Ohio State University, Columbus, Ohio, United States of America; 7 Department of Anthropology, SUNY Oneonta, New York, United States of America; 8 Center for the Advanced Study of Human Paleobiology, Department of Anthropology, The George Washington University, Washington District of Columbia, United States of America; 9 Centro Nacional de Investigación sobre la Evolución Humana, Burgos, Spain; 10 School of Anthropology and Conservation, University of Kent, Canterbury, United Kingdom; 11 Department of Sociology, College of Liberal Arts, Purdue University, West Lafayette, Indiana, United States of America; 12 Mathematics and Statistics Research Group, University of the West of England, Bristol, United Kingdom; University of Sao Paulo: Universidade de Sao Paulo, BRAZIL

## Abstract

Accentuated Lines (ALs) in tooth enamel can reflect metabolic disruptions from physiological or psychological stresses during development. They can therefore serve as a retrospective biomarker of generalized stress exposure in archaeological and clinical research. However, little consensus exists on when ALs are identified and inter-rater reliability is poorly quantified across studies. Here, we sought to address this gap by examining the reliability of accentuated (AL) markings across raters, in terms of both the presence versus absence of ALs and their intensity (HAL= Highly Accentuated, MAL= Mildly Accentuated, RL= Retzius Line). Ratings were made and compared across *observers* (with different levels of experience) and *pairs of raters* (who agreed on AL coding through consensus meetings) (N = 15 teeth, eight observers). Results indicated that more experience in AL assessment does not necessarily produce higher reliability between raters. Most disagreements in intensity ratings occurred in categories other than HAL. Furthermore, when AL assessment was performed by pairs of raters, reliability was significantly higher than individual assessments (Gwet’s AC1 = 0.28 to 0.56 for line presence assessment; Gwet’s AC1 = 0.48 to 0.64 for line intensity assessment). Based on these results, we recommend a workflow called IRRISS (**I**mproving **R**eliability and **R**eporting **I**n **S**coring of **S**tress-markers) to increase rigor and reproducibility in histological analysis of dental collections. The introduction of IRRISS is well-timed, given the surge in studies of teeth occurring across anthropological, epidemiological, medical, forensic, and climate research fields.

## Introduction

Early-life stress exposure is a known risk factor for health issues later in life, including physical and psychiatric disorders across the life course [1–4]. Indeed, exposure to stressors during childhood, whether of a physical (e.g., illness, nutritional deficiency) or psychological nature (e.g., abuse, neglect, other trauma) can increase the risk of childhood- and adult-onset disorders by twofold or more [2,5]. Identifying children who were exposed to early life stress, particularly during prenatal and perinatal life (two potentially sensitive periods when stress exposure may have more enduring effects), remains a central challenge for clinical researchers and medical specialists [2,6,7]. The search to identify more objective and reliable ways of measuring the timing and severity of stress exposure during early life led Davis and colleagues [8] to propose the use of deciduous teeth for this purpose, given that teeth retain a permanent record of somatic growth – and growth disruptions related to stress exposure – in their microstructure [8, 9].

**Fig 1:**
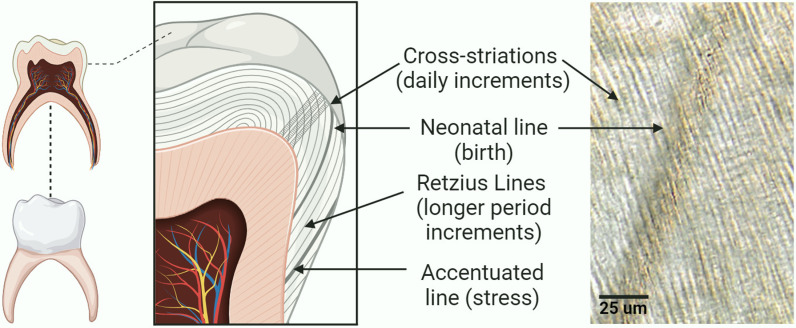
Schematic overview of dental microstructure. Daily incremental rhythm, near-weekly rhythm, and stress-related increments are visible in histological sections of tooth enamel (Figure made by the authors using BioRender®).

In brief, tooth enamel contains distinct types of incremental growth patterns in its microstructure, a characteristic shared with other biological structures and organisms, including bones, shells, and wood (Fig 1) [10–12]. Short-period increments (known as cross striations) follow a daily rhythm corresponding to the circadian rhythm in enamel matrix formation [10,13]. Long-period increments, known as Retzius lines [10,14], follow a circaseptan (nearly weekly) formation rhythm or periodicity [15–18]. Retzius line periodicity reflects oscillations of biological rhythms specific to each individual’s circaseptan rhythm, often ranging from 6-9 days [15,16,19]. These incremental growth patterns are evident in both deciduous and permanent teeth and reflect an individuals’ biorythm and somatic growth rate [12,14,19].

Although dental development is under strong genetic control, the cells that deposit the enamel matrix (ameloblasts) are sensitive to disruptions of homeostasis [9,12,13,20]. Systemic disruptions to homeostasis can produce various dental defects [21–26], as well as an alteration to the enamel and dentine microstructure [27–30]. Accentuated Lines (ALs), also known as stress lines or Wilson bands [9,13,20,21,23–25], are one major type of alteration; these alterations run parallel to Retzius lines and are often irregularly spaced. ALs can appear thicker or darker than the normal rhythmic Retzius lines, and can often be visible deep into the enamel (rather than Retzius lines, which in thin sections are mainly visible closely to the outer enamel surface). Similar to Retzius lines and daily increments, ALs can be visualized in teeth using conventional white light microscopy (12, 23). Although the aetiology of these lines is an ongoing line of scientific enquiry, scientists often use the term ‘stress lines’, as the timing of AL formation often coincides with a documented event or period of some type of stressor, including life history or transitional events (e.g., mode and duration of delivery [31–34], menarche, and parturition [35–37], health conditions (e.g., nutritional deficiencies, fevers and injuries [9,28,38–40] medicine administration [19,41]), or psychological stressors (e.g., traumatic life events; stressful life events [35,39,42,43].

Because enamel does not remodel or regenerate after formation, these ALs are permanently recorded and can serve as a retrospective marker of stressor exposure [14,44]. Given that deciduous teeth start developing in utero and continue forming during the first years of life, overlapping with known sensitive periods for brain development [45–47], ALs visible in deciduous teeth could provide important retrospective data about stress exposure in utero and during early life that may impact later health. ALs therefore have a high diagnostic potential as a biomarker in medical [8,42], forensic [48] and anthropological studies [44] to identify people and populations exposed to growth disruptions caused by early life stress exposure.

**Fig 2:**
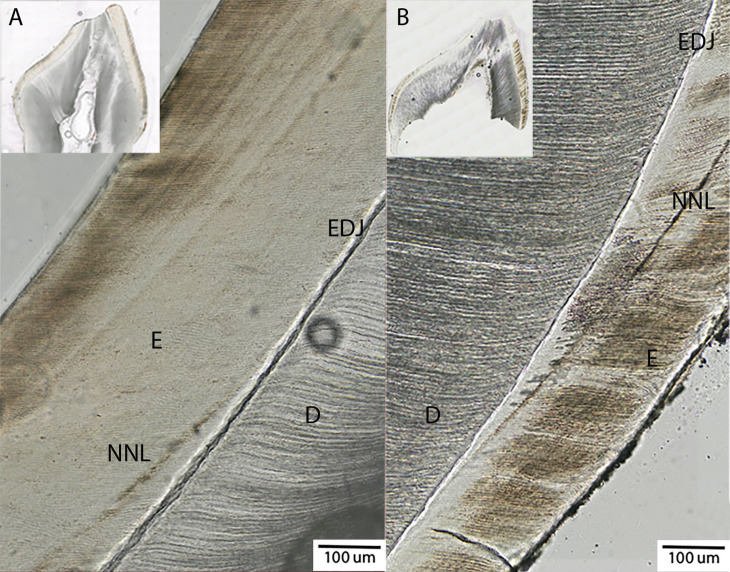
Example of variation in dental microstructure between two deciduous tooth sections shown at x20 magnification. NNL = Neonatal line. E = Enamel. D = Dentine. EDJ =  Enamel Dentine Junction. The NNL is generally clearly distinguishable in tooth thin sections but can vary in its manifestation. For example (A) shows the NNL as a wide and diffuse line. In contrast, (B) shows the NNL as sharp and narrow. Other Als in deciduous teeth are rarely as clear and unambiguous as the NNL. In some cases, these other ALs cannot be distinguished from regular incremental deposition lines (i.e., Retzius lines) with certainty.

The variation in AL appearance, whether dark or broad, diffuse to narrow, and sharp, to bright (Fig 2), may be influenced by many factors, such as the timing, type, and intensity of the stress exposure, the tooth type studied (incisor vs. molar), the position of the line within the tooth (cuspal vs. lateral enamel), as well as sample preservation, preparation techniques and equipment used for assessment [20,49–51]. Thus, ALs are measured on a continuous scale, ranging from mildly to highly visible [9,30,52–55]. The most notable AL is the neonatal line (NNL), which corresponds to physiological changes associated with birth [56]. Scientists observed the presence of the NNL as early as 1936 [34,57]. Since then ALs found their way particularly in fundamental studies on dental anatomy, human evolution, and biological anthropology [12,14]. However, in recent decades, there has been a surge in efforts to incorporate ALs in teeth as retrospective archives of stress exposures. Many fields of study, including forensics [58–61], medicine [8,42,62–65] and climate science [42,66–69] have measured ALs in data collection efforts. One reason for the increasing popularity of studying ALs is that they are thought by some to provide an *objective* biomarker that can retrospectively assess early-life stress exposure [8]. However, the wide variety in AL appearance makes assessment *subjective* and therefore challenging.

In this regard, AL assessment is no different from histological analysis in medical or forensic research, which often requires the interpretation of subjective variables and the expression of features and lesions [70, 71]. Such subjective variables can range from dermatological lesion scorings of size, shape, colour, and border elevation, [72] and oncology studies relying on visual tumour grade classification [73]. Differences in study measurements between raters may therefore vary based on the level of disagreement or error introduced by inconsistencies between data collectors [74]. Between-rater agreement in feature assessment is generally thought to improve by outlining clear scoring criteria and having more experienced raters [74].

Although researchers have categorized ALs based on their length, intensity, and structure irregularity [9,20,29,55], it is unclear how much consistency exists between dental histologists for both the presence and intensity of AL assessment. In a citizen science project called ‘Diary of a Tooth,’ Gamble and Ferrell [75] examined variation in how members of the public – or citizen scientists – identified an AL. Preliminary findings from this project revealed a tendency for consensus between raters when lines were highly prominent, but underscored the challenges of assessing ALs, particularly for untrained participants.

Most studies on AL assessment mention that line counts were performed multiple times by the same rater [64,76–78] or by two independent raters who calibrated their results [29,37,52,79]. When multiple teeth from the same individual are included in studies, ALs can be matched between teeth, because portions of crowns that form during the same time can register the same AL pattern [29,80–82]. In such cases, the presence of ALs between time-overlapping teeth can serve as a confirmation of AL presence and therefore improve the reliability of AL presence. However, it is unclear how much AL scoring differs between raters and how much assessment deviates when performed by different research groups, where training styles and experiences may differ, especially when assessments are made on single teeth without calibrating ALs across a dentition. Varying disagreements between raters could be problematic both for archaeological datasets (often with sample sizes of n < 30) and for clinical data collection efforts with potentially thousands of samples, as the robustness of data is heavily dependent on its reliability. Because AL measurement has increased substantially as a high-value biomarker for retrospective stress exposure, it is crucial to assess the reliability of AL assessments so rigorous protocols on correct implementation can facilitate accurate, reliable, and meaningful data collection.

In this study, we addressed these issues by assessing inter-rater reliability in ratings of ALs between eight biological anthropologists from different research groups, with varying levels of experience in dental histology. Our goal was to assess inter-rater reliability between *individuals*, examine how inter-rater reliability varies when assessing the intensity of AL manifestation, and quantify the level of reliability between *pairs of raters*. To achieve these goals, we first determined the reliability of AL markings between individual raters with varying levels of experience, asking:

- (1a) How reliable are ratings between individual raters when evaluating the *presence* of AL?
- (1b) How reliable are ratings when assessing the *intensity* of the AL?

Second, we examined the reliability of pair ratings in comparison to individual ratings, asking:

- (2a) How reliable are ratings between two individuals assessing the presence and intensity of AL when provided measurement guidelines based on AL length?
- (2b) Do these pair-derived consensus ratings yield higher reliability than ratings across individuals?

By answering these questions, we aimed to critically evaluate if current data collection practices of AL assessment are sufficient to address the research questions being posed across diverse disciplines, and transparently examine the potential need for change in methodological standards.

## Materials and methods

### Rater profiles

Eight biological anthropologists (SAML, MLL, KG, MOH, KMG, JAG, RJF, DGS) with experience in dental histology assessed ALs for this study. Each rater completed a questionnaire, providing detailed information about their level and type of experience in AL assessment (Table 1). We categorized rater experience levels through a combination of their level and type of experience in relation to the objectives of this study. As shown in the ‘AL identification experience’ category, all raters had AL identification experience, defined as having counted their presence in tooth slides, and having observed them while performing other histomorphometric analyses of dental tissues (such as measurements of daily and longer period increments). Four of the raters had experience with AL position measuring, defined as having specific experience with measuring the position of accentuated lines within the enamel and calculating the timing of their formation in relation to the age of the individual, rather than merely counting or observing ALs.

**Table 1:** Overview of rater profiles.

Rater ID	Affiliated lab	Seniority level	Dental histology experience (years)	# dental histology slides assessed	AL identification experience	Experience with AL position measures	Published on AL measures in deciduous teeth
DGS	OSU	Senior	12 +	500 +	Yes	No	No
RJF	N/A	Senior	12 +	500 +	Yes	Yes	Yes
SAML	MGH/HMS	Mid	7-12	200-500	Yes	Yes	Yes
KMG	GW	Mid	7-12	200-500	Yes	Yes	Yes
JAG	Manitoba	Mid	7-12	<200	Yes	Yes	No
KG	OSU	Junior	3-5	<200	Yes	No	No
MLL	MGH/HMS	Junior	5-7	<200	Yes	No	No
MOH	KENT	Junior	5-7	200-500	Yes	No	No

All raters were highly familiar with the concept of ALs and had previously worked on thin sections where ALs were visible. While some raters specifically focused on stress assessment through ALs in their research (SAML, KMG, KG, RJF, JAG) [52,53,76,81,83–85], others primarily conducted counts or used ALs as reference points for calculating crown formation times (GS, MOH, MLL) [86–88]. Four of the raters had experience measuring the position of ALs; of those, three had experience specifically measuring and calculating the position of ALs in deciduous teeth (versus permanent teeth) (Table 1). Based on the data from the questionnaires, we established three main categories of experience and seniority level: senior, mid, and junior (Table 1).

### Sample selection and slide sharing

In this study, we focused on a subset of exfoliated primary teeth donated to an ongoing modern cohort study (called the Stories Teeth Record of Newborn Growth, or STRONG, Principal Investigator: E.C. Dunn), as there is a clear demand for the analysis of large numbers of primary teeth in medical research [8,42,64,89,90]. STRONG is a quasi-experimental research study designed to investigate if children’s teeth record evidence of their mother’s exposure to a calendar-dated major stressful life event: the Boston Marathon bombings and manhunt event events. STRONG participants (n = 285 mother-child pairs of children born between April 1, 2012, and November 4, 2013) were identified through hospital- and community-based methods and were reimbursed for donating up to 5 teeth to the study. Ethical approval for the STRONG study was obtained from the Mass General Brigham Institutional Review Board (IRB) on December 16, 2019 (protocol ID 2019P003570). Recruitment began on December 18, 2019, and ended on March 28, 2022. The collection of exfoliated primary teeth is ongoing. Informed consent for using human specimens and data collected via questionnaires and electronic medical records was obtained from parents via written informed consent and child verbal assent.

For the current study, we selected samples of single-cusped deciduous teeth (incisors and canines) for which both prenatal and postnatal enamel was still preserved, to allow for enough enamel to be present for AL assessment. ECD, SJS and AGH had access to information that could identify individual participants during and after data collection, whereas all eight raters (Table 1) were blind to all phenotype data from the STRONG study, including participants’ level of marathon/bombings stress exposure. All research adhered to the Declaration of Helsinki and the Health Insurance Portability and Accountability Act, as well as standards of reporting.

All teeth in the STRONG collection were embedded in polyester epoxy resin. We cut labial-lingual sections with a slow-speed diamond-wafering blade precision saw (Isomet 4000). We fixed the cut sections on petrographic microscopy slides, and subsequently ground and polished each using a graded series of grinding and polishing pads reaching a final thickness of around 100um [91, 92]. We imaged the sections with an Olympus BX61 upright brightfield microscope coupled with an Olympus VS120 slide scanner and Orca R2 monochrome (16-bit) camera with plan apochromatic objectives. Images of the tooth slides assessed in this study are available via Harvard Dataverse (https://doi.org/10.7910/DVN/PBIOZ1).

One rater (SAML) did a general assessment of a subset of the STRONG collection and selected 15 tooth slides from 15 different individuals, aiming to include slides with evidence via ALs of prenatal stress, postnatal stress, or both. The goal was to avoid a high percentage of slides without ALs to prevent artificially inflated reliability between raters indicating an absence of ALs. All raters reviewed all 15 slides without any knowledge of individual life histories.

As participating raters were from multiple research groups, countries, and continents, we shared images in.vsi format as pyramidal files using QuPath, a cross-platform, open-source software for digital histology and whole slide image analysis [93]. Virtual pyramidal slides allowed raters to examine each slide using 20x and 40x objective magnifications, enabling them to freely zoom in and out, adjust contrast, and thoroughly explore histological features. The intention was to replicate the experience of traditional transmitted light microscopy with physical slides, rather than restricting access to a single fixed image of limited resolution.

### Data collection – two approaches

We devised two separate approaches to answer our research questions. Each approach was designed to mimic data collection practices commonly described in published work on ALs (Table 2). In Approach 1, to address research questions 1a and 1b, we provided the eight raters with a first set of 10 slides. We recorded how each rater individually marked and scored AL presence and intensity. We asked every rater to mark the neonatal line in the enamel in each micrograph and identify other ALs they saw as either highly accentuated (HAL) or mildly accentuated (MAL) in the enamel and the dentine. All raters were given guidelines on what constitutes a HAL or MAL (Table 2), based on the length of the features [9]. Scoring criteria were based on intensity, following commonly used terminology in the literature (see details in Table 2). In Approach 2, to address research questions 2a and 2b, we provided a subset of six raters with a second set of 5 slides, and partnered each rater, creating 3 pairs of raters. We paired raters strategically to ensure objectivity, specifically selecting pairs based on their limited track-record of working together; this approach minimized potential biases arising from familiarity with each other’s scoring styles, and thus was aimed at enhancing the reliability of the study results. We asked each rater to individually identify and rate ALs in the set of slides, and subsequently discuss their ratings of these slides with their partner while jointly assessing the same slides to provide a consensus pair rating.

**Table 2:** Definitions of AL scorings for Approach 1 and Approach 2.

	Definitions of AL for Approach 1 Reliability of AL markings between raters with varying levels of experience	Definitions of AL for Approach 2 Reliability of individuals vs. pair ratings
	Based on intensity, following generally used terminology in literature	Based on the length of the line visible throughout the enamel area
Category 1: HAL	A standard definition of AL: Feature occurring in response to growth-disrupting physiological stress, and appearing in all concurrently forming teeth. They run parallel to ‘normal’ striae of Retzius and are often irregularly spaced. They tend to appear thicker, darker, and/or can be visible deeper into the enamel thickness compared to regular striae of Retzius [14,20,38,77,94].	The AL is visible for at least 75% of its length from the Enamel Dentine Junction (EDJ) to the Outer Enamel Surface (OES), and is significantly different in appearance from the surrounding matrix [9,20,29,37,51,53,64,78,79,81,94,95]
Category 2: MAL	Those ALs which are less intensely accentuated and more doubtful (in line with descriptions noted in [30,52,53].	At least 50-75% traceable along the enamel forming front from the EDJ to OES and noticeably different from the surrounding matrix [30,55]

### Data merging

One rater (SAML) compiled all the ratings and cross-compared micrographs to match all identified HAL and MAL scorings between the raters, giving the identified lines a code (Stress 1, Stress 2) to allow calibration of the marked increments between all raters. When a rater did not score a line as accentuated where another did, we classified that raters’ absence of marking as a third category ‘Retzius line’ (RL), referring to the rhythmic growth lines representing the normal, incremental pattern of enamel deposition [14]. In other words, if rater 1 identified an AL, but rater 2 did not, then rater 2’s identified line was coded as RL for the position of this feature, as they considered this feature a normal growth increment rather than an AL. Unlike when using a citizen science approach, where raters have limited or no experience [75], we considered each rated line to be a growth mark (whether accentuated or not). Deciduous anterior teeth only rarely show clear, consistent Retzius lines throughout the enamel, unlike deciduous molars. Hence, we agreed to mark the third category as RL, as vaguely visible features in tooth enamel, if not accentuated, will likely be Retzius lines.

We created a dataset for analysis as follows. First, to assess the reliability in ratings for the presence (vs. absence) of an AL, ratings of MAL and HAL were collapsed into a single “AL” category. Second, to assess the reliability in ratings for the severity of the line, we analyzed the original scorings of MAL, HAL, and inferred RL categories. Because line visibility may be less sharply defined in dentine compared to enamel, and lines present in dentine may seem broader and darker [19,53], we rated all ALs marked in the dentine (n = 29) as HAL. Therefore, no intensity-related analyses were done for ALs in the dentine. Furthermore, we excluded ratings when raters marked a broader line as two separate lines (e.g., neonatal line doubling; n = 3) to avoid counting the same feature twice. Lastly, we excluded two cases where a rater marked a crack in dentine as an AL, which followed the direction of matrix deposition.

### Statistical test of inter-rater reliability

We used Gwet’s AC1 to measure reliability in identifying ALs between individuals and pairs. Gwet’s AC1 was used rather than traditional kappa statistics (e.g., Cohen’s kappa) because our data could be sensitive to the “Kappa’s paradox” [96] where high levels of reliability are artificially and substantially reduced due to a high prevalence in one category of measure (i.e., Retzius Lines). Gwet’s AC1 statistic is more robust to high prevalence in one category by utilizing an expected disagreement rate rather than an agreement rate [97, 98]. Because kappa statistics are more widely used and have readily available thresholds for interpretation (S1 Table), Light’s kappa was also calculated and shown in S2 Table. However, it must be noted that Gwet’s AC1 is generally higher than kappa statistics. Although thresholds for kappa statistics are sometimes similarly applied to Gwet’s AC1, recent work has called for caution with direct comparisons [98]. As there are no new thresholds created for the correct interpretation of Gwet’s AC1 yet, we follow the example of others [99] by reporting both kappa statistics and Gwet’s AC1 for comparison. Keeping in mind that threshold classifications are subject specific, what can be considered “good” interrater reliability in one field, for example psychology or sociology, might be unacceptable in medical or forensic contexts [74,100]. Hence, kappa thresholds should be considered as a guideline for interpretation rather than fixed unarguable categories.

For Gwet’s AC1, we calculated unweighted AC1 for presence/absence. For intensity ratings, which are ordinal, it was appropriate to calculate the linear weighted AC1. Weighted reliability coefficients are employed when it is necessary to account for varying magnitudes of difference in ratings (i.e., level of intensity), thereby giving more importance – or weighting – to larger differences (i.e., HAL vs. RL). Our categorization of AL marking (HAL, MAL, or RL) was as an ordinal variable from least (not) accentuated (Retzius Line, RL) to most accentuated (HAL). Therefore, disagreement between raters on RL vs HAL ratings was penalized more than disagreement between the RL/MAL and MAL/HAL ratings when using a weighted AC1. While ALs exist on a spectrum of intensity rather than fixed categories, we treated a disagreement in the choice between RL and MAL as linearly equivalent to a disagreement between the MAL and HAL choices. All statistical analyses were conducted in R using the irr and irrCAC packages [101–103].

## Results

### Approach 1: Reliability of AL markings between raters with varying levels of experience

**Fig 3:**
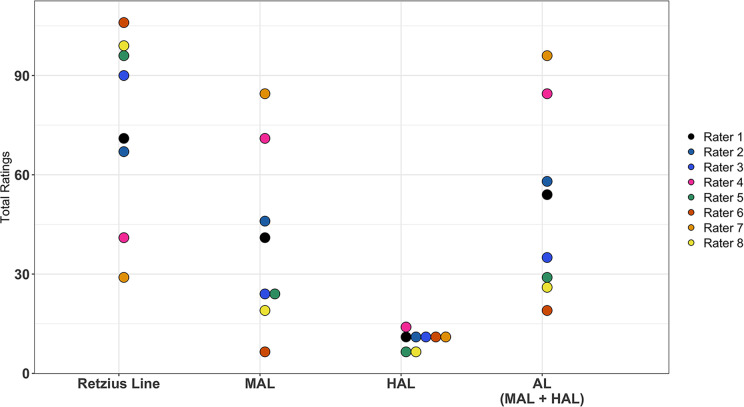
Total ratings of features reported by eight raters in ten histological slides for Approach 1. The x-axis indicates the type of histological feature rated. The y-axis indicates the frequency count of the feature for a given rater. Each color represents one rater, with color choice being random. Raters who were most inclusive in their AL ratings (e.g., rater 6) had the lowest number of Retzius lines reported, meaning they interpreted the highest number of features as accentuated. Those who were the most stringent in their assessment with the lowest number of ALs (e.g., Rater 7, in orange) had the highest number of Retzius lines, as they more seldomly deemed features to be accentuated.

Combined, the eight raters identified a total of 125 features across the first 10 slides, comprising both HAL, MAL and RL (Fig 3). Raters who had the highest number of ALs (MAL or HAL) in their assessment had the lowest number of RLs recorded and vice versa. Raters individually observed between 1 to 6 HALs. The observed total counts for MAL ratings were much more varied across raters (ranging from 7 to 84).

The average line presence reliability between all raters using Gwet’s AC1 was 0.31 (**Table 3**, with additional details plotted by reliability coefficient in S3 Fig). Taking experience into account, junior raters had the highest between-rater reliability (AC1 = 0.66), compared to mid-career (AC1 = 0.20) and senior raters (AC1 = 0.15).

**Table 3:** Gwet’s AC1 scores related to results of objective 1 on the reliability of raters according to level of experience and line intensity.

	Line Presence Reliability: Gwet’s AC1 (CI)	Line Intensity Reliability: Unweighted Gwet’s AC1 (CI)	Line Intensity Reliability: Linear Weighted Gwet’s AC1 (CI)
All Raters (n = 8)	0.31 (0.25,0.36)	0.44 (0.39, 0.49)	0.60 (0.55,0.65)
Seniority	–	–	–
Senior (n = 2)	0.15 (-0.01,0.31)	0.30 (0.18, 0.43)	0.51 (0.41,0.60)
Mid (n = 3)	0.20 (0.10,0.30)	0.37 (0.29,0.46)	0.55 (0.47,0.62)
Junior (n = 3)	0.66 (0.57,0.76)	0.70 (0.62.0.78)	0.80 (0.73,0.86)
Tissue (All Raters; n = 8)	–	–	–
Prenatal Enamel	0.32 (0.22,0.41)	0.46 (0.39,0.52)	0.64 (0.58,0.70)
Postnatal Enamel	0.30 (0.21,0.38)	0.42 (0.35,0.49)	0.57 (0.50,0.65)
Dentine	0.32 (0.20,0.45)	–	–

CI =  95% Confidence Interval.

The average weighted reliability of AL intensity ratings between all raters was moderate (AC1 = 0.60). When considering experience level, senior and mid-raters had similar reliability (AC1 = 0.51 and AC1 = 0.55, respectively), whereas junior raters had higher reliability (AC1 = 0.80). Hence, even though reliability ratings were higher for weighted measures compared to unweighted measures, the same pattern persisted where junior ratings had higher reliability than the other groups. The reliability of AL presence and intensity ratings remained relatively consistent across tooth regions, including prenatal enamel, postnatal enamel, and dentine, meaning that tissue type did not seem to influence rating reliability.

Disagreement between all raters was highest when deciding if a feature was either mildly accentuated or not accentuated at all; 92 out of all 125 counted ratings were classified as either MAL or RL (73.6% of all ratings). It was far less common to have a full disagreement on AL assessment between raters (meaning where one rater indicated a line was HAL, while a second rater labelled it MAL, and a third labelled it RL (N = 22; 17.6%). Notably, 17 (77%) of these ‘cross-rater disagreement’ cases were due to a single rater differing in their assessment from the other raters. For example, when seven out of eight raters all agreed the line was an AL, either as HAL or as MAL, one rater marked an absence of AL altogether (RL). There were also instances of the opposite, where all agreed on the absence of a feature while one rater marked a HAL. In 4 cases, two raters differed from other raters (e.g., 6 raters marked HAL, 1 rater marked MAL, 1 rater marked RL).

Unweighted reliability assessments of AL intensity between all raters and tooth regions were lower than weighted AC1 results, as expected; this difference was because the weighted reliability score emphasized larger disagreements (HAL vs RL). As the largest amount of disagreement between raters were less “severe” (i.e., MAL vs. RL, rather than RL vs. HAL), weighed AC1 results ratings provide a better statistic to evaluate levels of agreement on highly accentuated feature presence.

### Approach 2: Reliability of individuals vs. pair ratings

In the second dataset with stricter defined rating guidelines, as shown in **Table 4**, we saw a similar overall pattern of results, where reliability of average line presence (versus absence) between all individuals was lower than the reliability of line intensity (with additional details plotted by reliability coefficient in S4 Fig).

**Table 4:** Overview of Objective 2 results on the reliability of individual vs pair ratings.

		Line Presence Reliability Gwet’s AC1 (CI)	Line Intensity Reliability Unweighted Gwet’s AC1 (CI)	Line Intensity Reliability Linear Weighted Gwet’s AC1 (CI)
All raters (n = 6)		0.28 (0.13,0.42)	0.45 (0.33,0.58)	0.48 (0.33,0.64)
Within pairs				
Pair 1 (n = 2)	KG + MLL Junior + Junior	0.43 (0.08,0.78)	0.40 (0.09,0.72)	0.41 (0.05,0.77)
Pair 2 (n = 2)	RJF + JG Senior + Mid	0.47 (0.13,0.80)	0.51 (0.21,0.82)	0.56 (0.26,0.86)
Pair 3 (n = 2)	SAML + MOH Mid + Junior	0.23 (-0.15,0.62)	0.38 (0.07,0.70)	0.41 (0.08,0.75)
Between pairs after Consensus (n = 3)		0.56 (0.32,0.79)	0.59 (0.37,0.81)	0.64 (0.43,0.86)
Difference of reliability score after consensus^*^		+0.28	+0.14	+0.16

CI =  95% Confidence Interval;

* + values in the last row indicate an improvement in score.

When looking at the reliability between two paired raters, there was distinct variation. Whereas two pairs (Junior +  Junior; Senior +  Mid) had fair reliability, one pair of Mid +  Junior had the lowest reliability. This result is partially in line with the previous results that the average reliability between junior raters was high, while raters with deviating levels of experience can have lower reliability. Differences between weighted and unweighted line intensity reliability coefficients were minimal for all rater groupings. These results show that even though individuals within a pair could have individually diverging ratings, after a consensus meeting their final data collection became more comparable to those of other pairs, resulting in a higher reliability score across pairs. Compared to individual assessments, pair reliability doubled for line presence (from Gwet’s AC1 0.28 to 0.56) and increased by 33% for line intensity assessment (from Gwet’s AC1 0.48 to 0.64).

**Fig 4:**
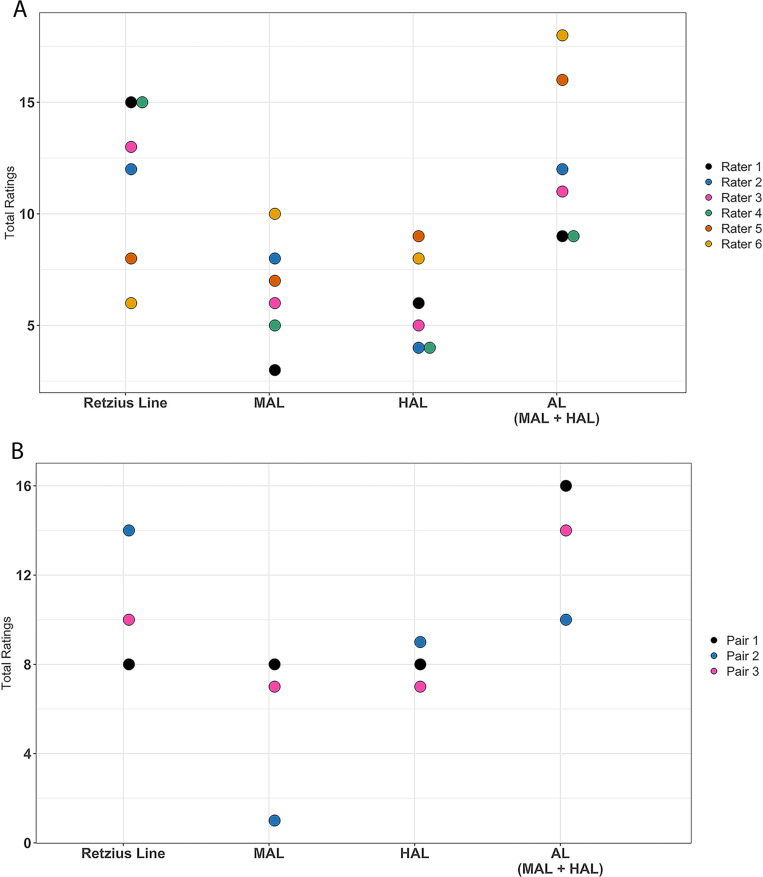
Total ratings of AL types reported by six raters in five histological slides in Approach 2. In both panels, the x-axis indicates the type of histological feature rated, and the y-axis indicates the frequency of the feature for a given rater. (A): Total ratings of AL types for individual raters before consensus, with each color presenting one rater, listed in random order. (B): Total ratings of AL types for three pairs of raters after consensus, with each color presenting one pair in random order. Those raters or pairs who were most inclusive in their AL ratings (e.g., (A) rater 6, (B), pair 1) reported the lowest number of Retzius lines, meaning they interpreted the highest number of features being accentuated compared to the other raters.

The total number of HAL observed among individual raters was 4 – 9, while the observed counts for MAL ratings across raters was 1–10 (**Fig 4**). After team members made consensus ratings, the total number of HALs observed among teams was 7 – 9, with the observed counts for MAL still having higher variance across teams (1 – 8). Visual representations of the ratings of team members on their histological sections are shown in the S1 and S2 Figs.

## Discussion

Three main findings emerged from this study. First, in individual assessments of AL evaluations, with no quantifiable rating guidelines, raters with less experience showed higher reliability between themselves compared to groups of raters with more experience in dental histology. Second, consensus ratings between pairs of raters had higher reliability than individual ratings. Third, MAL ratings were possibly less reliable than HAL ratings, both individually and in pair assessments.

The findings of our study are both consistent and inconsistent with prior literature. AL markings are known to be challenging to assess due to the high level of variation in their appearance, and therefore, little consensus exists on how they should be scored [20,29,55]. Our study examined and confirmed low consensus in individual rating approaches. The raters on our team, despite having a high level of familiarity with ALs, seem to have different approaches to AL identification, even when given the same scoring criteria. These findings are notably consistent with previous work showing similar challenges in AL assessment in the lay public [75]. Thus, both experienced and inexperienced raters have challenges in AL assessment.

Our finding that senior raters can have lower reliability than junior raters challenges statements that greater experience can lead to higher agreement and reliability in image analysis assessments [74,104,105]. Our results suggest that providing more training and experience with AL assessment may not guarantee improvement in reliability in AL evaluations. We suspect differences between senior and junior raters may reflect different approaches to ‘inclusiveness’ in data collection. That is, the raters in our study were from various laboratories, and although they collaborate with each other and overlap in research activities, their training and experience occurred in different research groups. Moreover, we found diversity in how inclusive raters were in their markings, especially when it came to the milder features (i.e., MAL). Some individuals, mainly junior raters, were more prone to exclude subtle features, whereas others were inclined to add them into the dataset as possible stress markers. Faced with uncertainty, junior raters may have decided to leave subtle features out rather than include them. Senior raters, however, having seen more variation in the manifestation of ALs, seem to have varying approaches towards including vs. excluding milder features.

Our finding that pair ratings were more reliable than individual ratings may be due to several factors. For one, consensus meetings may encourage team members to adhere to scoring criteria. That is, teams may be less likely to include doubtful features in their joint ratings, thus following scoring criteria more stringently. Team members also commented that consensus meetings encouraged them to be more vigilant. They noted ALs that might be missed by a single rater, and sometimes even prominent ALs, were less likely to slip past their notice when two raters were assessing the slide together. Furthermore, in Approach 2, the differences between weighted and unweighted line intensity reliability coefficients were minimal for all rater groupings. Similarities in these reliability coefficients suggest that given additional guidelines for ratings (as we did for Approach 2), the distribution of RL/MAL/HAL was more balanced, or raters had more consistent agreement and disagreement patterns across all three categories.

In addition to these three major findings, several other important findings emerged from this study. In both approaches, we found disagreements between MAL and RL were more prominent than MAL/HAL and RL/HAL ratings. Higher disagreement on subtle features may be a logical consequence of the most prominent lines being generally spotted by all raters, regardless of the level of experience and the individual scoring styles. These findings were also noted by Gamble & Ferrell’s 2019 citizen science project [75].

The disagreement on subtle markings might also relate to the tooth types we studied. Differentiating MALs from normal growth lines could be more challenging in deciduous anterior teeth than in deciduous molars or permanent teeth. In our experience, Retzius lines are rarely clearly visible in deciduous anterior teeth. Therefore, should several successive mild features be present in the tooth, it may be challenging to categorize such features consistently and reliably. We noticed such difficulties both in prenatal and postnatal enamel.

**Fig 5:**
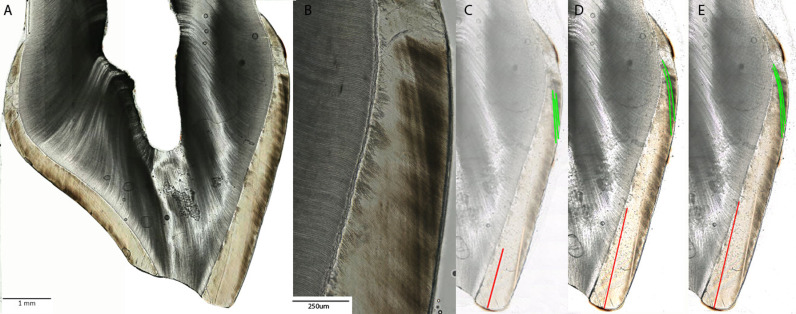
Example of a section used in this study demonstrating the difference between pair ratings. (A) Overview of a histological tooth section with the enamel in brown and dentine in dark black. The occlusal surface is facing down, with the dentine horn being exposed due to wear. The cervical enamel area shows clear ALs (red arrow). (B) Magnification of the indicated area with ALs from (A). (C, D, E) Ratings of pairs 1, 2, and 3, where each pair of two raters jointly marked the buccal enamel for AL presence and intensity. All pairs agreed on the NNL (red) and the presence of 1 MAL (off-white) as the first AL following the NNL. All pairs also marked the presence of multiple HALs in the cervical enamel (green) but had differences in how many HALs should be included in data collection.

A close examination of the micrographs also revealed that some disagreements were not as substantial as they might seem when considering isolated data points. For instance, in the cervical enamel area of a sample section (**Fig 5**), raters consistently observed multiple HALs closely positioned in sequence. Some raters identified these closely spaced lines as three HALs, while others noted two. Despite this discrepancy, all raters correctly identified a line at the exact location within the specific tooth. This discrepancy in the number of identified HALs led to a statistically significant difference in ratings, with one HAL being classified as not accentuated by others (RL). However, the consensus among raters suggests a common understanding of the presence of an AL at the specific timeframe of enamel formation of this specific tooth. It is important to emphasize that had the individual to whom this tooth section belongs indeed experienced a stress event during cervical enamel formation – and the research question was “Does the exposure to a stress event correlate with the formation of an AL?” – all raters would have indeed identified ALs correlating with the timing of the stress event. Therefore, the use of teeth as a stress exposure biomarker would not be hampered by a difference in data collection among raters. The example in Fig 5 also hints at a phenomenon cognitive psychology studies refer to as ‘visual clutter’ or visual crowding’, wherein people’s perception of individual features or items becomes more challenging when the items are grouped together rather than when each item is presented in isolation [106–108]. Although we did not specifically investigate this cognitive bias in our study, it is plausible the reliability of identifying ALs is lower when tooth sections exhibit a high number of closely spaced accentuated lines compared to those with accentuated lines presented in isolation.

Our study’s approach has several strengths. First, we had a large group of specialists from different organizations partake in data collection. Traditionally, studies on ALs only have only one or two histology specialists, generally from the same group or lab, performing the assessment. By combining data from multiple groups, we obtained a good representation of different styles and approaches to inclusiveness of feature assessment. Second, we analyzed dental samples from participants enrolled in a modern study, rather than selecting samples from an archaeological context, allowing our study findings to generalize to dental samples collected from other modern samples. Cohort studies often recruit a large number of participants to complete in-depth and detailed population-level analyses. Community-based studies like ours often recruit (agnostic to exposure or outcome status) a large number of participants from the community to complete in-depth assessments, with the goal of obtaining results that could be generalizable to the population. Numerous modern community-based as well as cohort studies (which follow participants recruited from the community or elsewhere over time) are also collecting deciduous teeth, including the Environmental influences on Child Health Outcomes (ECHO) study and the Adolescent Brain and Cognitive Development (ABCD) study in the United States; the Raton Perez collection in Spain; and the Tooth Fairy collection in France [109–112]. Our results may generalize to these and other studies seeking to characterize ALs in modern, population-based samples.

Our study also has some limitations. Due to the time-consuming nature of histological assessment, and the limited number of specialists available worldwide, we were constrained in the total number of slides we could evaluate. However, the sample size was comparable to samples often available in archaeological or anthropological studies. We also did not examine the effect of tooth type, sample thickness, or the use of polarized images of AL reliability scoring. In the future, reliability studies could be expanded to incorporate polarized images; such filters are regularly incorporated in data collection to increase visibility of histological features and may provide insights into the reliability of AL scoring. Future studies can also incorporate other tooth types, permanent dentitions, and samples from archaeological contexts, which have their own challenges, including the ability to detect ALs after the process of natural decay (or damage after long periods of deposition in archaeological samples). Finally, future studies could also focus on AL assessment in virtual dental histology, the inter-rater reliability between virtual dental histology with conventional histology, and where inter-rater reliability on tomography data could be affected by chosen data treatment and post-processing steps [113].

Our study also prompts consideration of several critical issues that need to be addressed when using teeth to reconstruct exposure to early life stress. The first issue concerns improving the reliability of ALs as markers of early life exposure, as our results clearly demonstrate that merely gaining more experience does not suffice. The second issue concerns the identification of what should count as an indicator of stress exposure. At the current state of research, it is not fully clear if milder ALs should be included in the search of stress biomarkers, or if only strongly observable features should be recorded. Studies choosing to include only the most prominent features would likely have higher reliability between raters. However, these studies may miss milder features, which could correlate with milder but still important physiological responses [9]. Depending on the research question, researchers may want to use teeth as biomarkers for exposure to repeated mild stressors. However, doing so comes with challenges. For instance, given the known challenges with AL assessment, histologists agree that published counts of ALs should be considered a ‘minimum’, rather than an absolute total [114], as not all stressors experienced by individuals result in ALs [81], nor will they all be spotted accordingly during analysis. Thus, conventional overarching criteria for identifying ALs may exclude subtler histological features [115], as those generally focus on what this study classified as HALs. Large cohort studies, where medical records and self-reported or records-based data on stressful life events can be matched with AL presence and intensity (as the STRONG study is doing) could increase understanding of how dental histology can be used as a retrospective record of mild and severe forms of stress exposure*.* A better grasp on the meaning and importance of these ‘lower threshold’ stress markers may also come from using multimodal approaches applied to MAL assessment (e.g., Raman spectroscopy) combined with life history data [51,63]. When large datasets become available, especially through modern cohort studies where deciduous teeth can be collected in large quantities with detailed individual life history data, deep learning might also improve our knowledge of the identification, classification and interpretation of accentuated line features [116–119].

Based on the reliability issues we uncovered in AL ratings, and to improve future data collection rigor and robustness, we recommend the following workflow to both increase reliability between dental histologists as well as assure robust data collection strategies and reproducibility (Table 5). We call this workflow the ‘IRRISS’ approach - **I**mproving **R**eliability and **R**eporting **I**n **S**coring of **S**tress-markers. Three core principles are at the heart of IRRISS: (1) Objective team assessment, (2) Transparency in data collection, and (3) Transparency in reporting reliability scores. These recommendations can be prospectively applied, meaning used to evaluate the quality of methods for studies performed in the future. These recommendations could also be considered retrospectively, when previously collected datasets on AL frequency and intensity are used as comparative datasets to newly acquired data using the IRRISS approach. Such a retrospective application is mainly relevant when single teeth were used for assessment [63,64,84], whereas studies including multiple teeth per individual and cross-matched accentuated lines within a dentition already have an additional strong reliability calibration [29,80,81]. Either way, users can indicate explicitly in their work the extent to which they followed the IRRISS approach.

**Table 5:** Recommended workflow for the IRRISS (Improving Reliability and Reporting In Scoring of Stress-markers) approach.

	Recommendation	Impact on scientist’s/lab’s workflow	Impact on the field
1	Have at least two specialists independently assess each histological section.	Common, although not standard practice in dental histology. Given that various research groups report two raters as their practice, this recommendation should not impact or cause additional work.	Ensures that collected data is a representation of the judgment of at least two specialists, thereby reducing the likelihood of outliers in data collection arising from individual preferences regarding the inclusiveness of features.
2	Report reliability scores (and which statistic was used) between the two independent scorings.	Raters should consult with a data analyst or statistician to determine the best way to measure reliability based on the number of raters and possible scores (e.g., weighted vs. unweighted, Cohen’s vs. Lights).	Provides readers with a quantified understanding of the ease of measurement and the reliability levels of accentuated lines within a specific tooth collection. Such data facilitates an assessment of the extent to which the obtained data may be useful as a comparative dataset, for general versus specific research needs in the future.
3	Publish the final consensus between the two specialists alongside the individual values.	For transparency, independent scorings published can be added to supplementary material. When reporting reliability scores, there is no minimum sample size recommended for reliability tests, but researchers should conduct a sample size calculation if they want to detect statistically significant reliability scores and be transparent in their reporting [100,120].	Greater transparency in reporting research findings will encourage others in the discipline to follow the example, creating an overall higher quality in data collection, reproducibility, and quality control among researchers.
4	Describe clearly what scoring criteria were used to define AL variables, how they are defined, and why those were chosen.	When research questions focus on severe stress exposures, raters might opt to only focus on the most distinct or visible accentuated lines. The choice of what criteria are followed should be clearly described in the methods section of a research paper. We recommend following guidelines on what defines a HAL based on [9], as their definition constitutes a quantifiable description of line length, increasing reliability among raters. When only HALs are marked in a sample, the chance to report on MAL frequencies is however missed.	Clear reporting in AL scoring recognizes that such measurements exist on a spectrum of intensity, offering clarity and insight into the essential criteria specialists consider for including features in datasets. Such reporting can also facilitate greater replication of study findings by allowing other researchers to use the same criteria.
5	Include lower threshold stress measurements, such as maximum 1 subcategory (highly accentuated (HAL) and mildly accentuated (MAL), whereby HAL is classified as above, and mild defined as by [30], when appropriate.	When research questions focus on stress exposure across a broader spectrum (e.g., mild to severe) including MAL as a subcategory will be useful. MALs scoring will likely have higher disagreement than HAL scoring. However, because MALs might be important for a specific research question, we recommend they be included in data collection, but only if issue 4 above is followed. More than 1 sub-category to differentiate milder accentuated lines will likely result in low reliability in rating assessment and is therefore not recommended. When including both HAL and MAL, we recommend using both weighted and unweighted reliability coefficients to better understand disagreement and agreement patterns.	Providing clarity and insight into specialists’ perspectives on establishing a threshold for categorizing features as ‘mild’ or ‘highly’ accentuated contributes to a more comprehensive interpretation of stress measurements in the field.
6	Publish in an open access repository all or a subset of micrographs, including those featuring ‘accentuated’ lines. We recommend at least 5 micrographs, or 10% of all slides assessed to be made available.	Open Science refers to making data and processes available to all, in order to create more rigorous and inclusive scientific investigation. Providing access to the micrographs used in analysis follows the recommendations of the FAIR guiding principles [121] which improve the Findability, Accessibility, Interoperability, and Reusability of data for replication purposes. The Harvard Dataverse is one suitable depository with options for researchers and organisations. It is open to the worldwide research community free for use.	Allowing readers to evaluate the original images of published datasets encourages transparency in reporting and in the long term fosters higher levels of agreement between individuals from different research groups and laboratories. It also allows research teams to use freely available data for comparison and replication purposes.

The assessment of inter-rater reliability, and subsequent improvement of reliability, is crucial if scientists want to implement the use of ALs as stress signatures in research. AL frequency and intensity assessment has thus far predominantly been used in anthropological [37,79,81,122,123], and archaeological contexts [20,29,52,76,78,84,95,124] to understand past lifeways, stress exposure, and populational health, on relatively small sample sizes (regularly ranging from N = 10 to 100). However, AL assessment of deciduous teeth is emerging in currently living population health and child development studies [64,89], as ALs have high potential as an objective biomarker for early life stress and adversity exposure [8,42]. Large cohort studies of currently living individuals that combine accentuated line data with detailed life history data will be able to offer insights into the correlation between accentuated line intensity and type of stress exposure and provide a deeper understanding of Goodman and Rose’s ‘Threshold model’ [9] on the relationship between stress intensity and defect formation.

## Conclusion

Our research suggests that experience level between raters does not meaningfully impact the reliability of AL data collection, and nuanced intensity ratings pose the greatest source of disagreement between raters. Our results underscore the importance of collaborative data collection and measurement. We make six recommendations for future research to increase scientific rigor and reproducibility, including working in pairs, sharing reliability scores, and providing visual aids for scoring approaches. These insights are vital for improving AL assessment reliability, fostering transparency among scientists, and advancing Open Science through data sharing. Although image analysis inherently carries a level of subjectivity that can be challenging to mitigate, clear reporting and transparency in decision-making following the suggested IRRISS guidelines would be an improvement over current best practices.

## Supporting information

S1 Fig Example of pair ratings where there was high agreement between pairs of raters. Panel A = Histological section of an incisor without markings. Panel B = consensus ratings of pair 1, 2, and 3. Red marking = NNL, off-white marking =  MAL.
(DOCX)

S2 Fig Example of pair ratings where there was low agreement between pairs of raters. Panel A = Histological section of an incisor without markings. Dark brown is the buccal enamel, black areas is dentine. Panel B = consensus ratings of pair 1, 2, and 3. Red =  NNL, green =  HAL, off-white =  MAL.
(DOCX)

S3 Fig Results from Approach 1 (GWETs AC1) plotted by reliability coefficient. (DOCX)

S4 Fig Results from Approach 2 (GWETs AC1) plotted by reliability coefficient. (DOCX)

S1 Table Interpretation of Kappa Statistic. Guidelines by Altman (1991), where a κ-value 0.20 is considered poor agreement, 0.21–0.40 fair, 0.41–0.60 moderate, 0.61–0.80 good and 0.81–1 very good. (DOCX)

S2 Table Overview results from Approach 1 with a further breakdown on type of experience and corresponding inter-rater reliability including kappa coefficients. (DOCX)

## References

[1] Giampetruzzi E, Tan AC, LoPilato A, Kitay B, Riva Posse P, McDonald WM, et al. The impact of adverse childhood experiences on adult depression severity and treatment outcomes. J Affect Disord. 2023;333:233–9. doi: 10.1016/j.jad.2023.04.071 37086798

[2] Dunn EC, Soare TW, Zhu Y, Simpkin AJ, Suderman MJ, Klengel T, et al. sensitive periods for the effect of childhood adversity on DNA methylation: Results from a prospective, longitudinal study. Biol Psychiatry. 2019;85(10):838–49. doi: 10.1016/j.biopsych.2018.12.023 30905381 PMC6552666

[3] Grummitt LR, Kreski NT, Kim SG, Platt J, Keyes KM, McLaughlin KA. Association of childhood adversity with morbidity and mortality in US adults: A systematic review. JAMA Pediatr. 2021;175(12):1269–78. doi: 10.1001/jamapediatrics.2021.2320 34605870 PMC9059254

[4] Lussier AA, Zhu Y, Smith BJ, Cerutti J, Fisher J, Melton PE, et al. Association between the timing of childhood adversity and epigenetic patterns across childhood and adolescence: findings from the Avon Longitudinal Study of Parents and Children (ALSPAC) prospective cohort. Lancet Child Adolesc Health. 2023;7(8):532–43. doi: 10.1016/S2352-4642(23)00127-X 37327798 PMC10527482

[5] McLaughlin KA, Greif Green J, Gruber MJ, Sampson NA, Zaslavsky AM, Kessler RC. Childhood adversities and first onset of psychiatric disorders in a national sample of US adolescents. Arch Gen Psychiatry. 2012;69(11):1151–60. doi: 10.1001/archgenpsychiatry.2011.2277 23117636 PMC3490224

[6] Knudsen EI. Sensitive periods in the development of the brain and behavior. J Cogn Neurosci. 2004;16(8):1412–25. doi: 10.1162/0898929042304796 15509387

[7] Lussier AA, Zhu Y, Smith BJ, Simpkin AJ, Smith ADAC, Suderman MJ, et al. Sensitive periods for the effect of childhood adversity on DNA methylation: Updated results from a prospective, longitudinal study. Biol Psychiatry Glob Open Sci. 2022;3(3):567–71. doi: 10.1016/j.bpsgos.2022.04.002 37519470 PMC10382690

[8] Davis KA, Mountain RV, Pickett OR, Den Besten PK, Bidlack FB, Dunn EC. Teeth as potential new tools to measure early-life adversity and subsequent mental health risk: An interdisciplinary review and conceptual model. Biol Psychiatry. 2020;87(6):502–13. doi: 10.1016/j.biopsych.2019.09.030 31858984 PMC7822497

[9] Goodman AH, Rose JC. Assessment of systemic physiological perturbations from dental enamel hypoplasias and associated histological structures. Am J Phys Anthropol. 1990;33(S11):59–110. doi: 10.1002/ajpa.1330330506

[10] Boyde A. The structure and development of mammalian enamel. London: The London Hospital Medical College; 1964.

[11] Dean MC. Growth layers and incremental markings in hard tissues; a review of the literature and some preliminary observations about enamel structure in Paranthropus boisei. Journal of Human Evolution. 1987;16(2):157–72. doi: 10.1016/0047-2484(87)90074-1

[12] Hillson S. Dental anthropology. Cambridge University Press; 2023.

[13] Ten Cate NA. Nanci A. Ten Cate’s oral histology: Development, structure, and function. St. Louis: Mo, Mosby; 2003.

[14] Hillson S. Tooth development in human evolution and bioarchaeology. New York: Cambridge University Press; 2014.

[15] Mahoney P, Miszkiewicz JJ, Pitfield R, Deter C, Guatelli-Steinberg D. Enamel biorhythms of humans and great apes: the Havers-Halberg Oscillation hypothesis reconsidered. J Anat. 2017;230(2):272–81. doi: 10.1111/joa.12551 27726135 PMC5244461

[16] Mahoney P, Miszkiewicz JJ, Pitfield R, Schlecht SH, Deter C, Guatelli-Steinberg D. Biorhythms, deciduous enamel thickness, and primary bone growth: a test of the Havers-Halberg Oscillation hypothesis. J Anat. 2016;228(6):919–28. doi: 10.1111/joa.12450 26914945 PMC5341586

[17] Smith TM. Experimental determination of the periodicity of incremental features in enamel. J Anat. 2006;208(1):99–113. doi: 10.1111/j.1469-7580.2006.00499.x 16420383 PMC2100182

[18] Antoine D. Evaluating the periodicity of incremental structures in dental enamel as a means of studying growth in children from past human populations. University of London, University College London (United Kingdom). 2001.

[19] Dean MC, Scandrett AE. The relation between long-period incremental markings in dentine and daily cross-striations in enamel in human teeth. Arch Oral Biol. 1996;41(3):233–41. doi: 10.1016/0003-9969(95)00137-9 8735009

[20] FitzGerald CM, Saunders SR. Test of histological methods of determining chronology of accentuated striae in deciduous teeth. Am J Phys Anthropol. 2005;127(3):277–90. doi: 10.1002/ajpa.10442 15584065

[21] Guatelli-Steinberg D, Ferrell RJ, Spence J. Linear enamel hypoplasia as an indicator of physiological stress in great apes: reviewing the evidence in light of enamel growth variation. Am J Phys Anthropol. 2012;148(2):191–204. doi: 10.1002/ajpa.21619 22610895

[22] Guatelli‐Steinberg D, Lukacs J. Interpreting sex differences in enamel hypoplasia in human and non‐human primates: Developmental, environmental, and cultural considerations. American Journal of Physical Anthropology. 1999;110(S29):73–126. 10.1002/(sici)1096-8644(1999)110:29+<73::aid-ajpa4>3.0.co;2-k 10601984

[23] Guatelli-Steinberg D. What teeth reveal about human evolution. Cambridge University Press; 2016.

[24] Goodman AH, Rose JC. Dental enamel hypoplasias as indicators of nutritional status. Advances in dental anthropology. 1991;5 225–40.

[25] Skinner MF. Meaningful measures of enamel hypoplasia: Prevalence and comparative intensity of developmental stress. Am J Biol Anthropol. 2023;180(4):761–7. doi: 10.1002/ajpa.24699 36790765

[26] Temple D. The mother-infant nexus revealed by linear enamel hypoplasia: chronological and contextual evaluation of developmental stress using incremental microstructures of enamel in late/final jomon period hunter-gatherers. In: Nowell A, Kurki H, Gowland R, Halcrow S, editor. The mother-infant nexus in anthropology: small beginnings, significant outcomes. 2020. p. 65–82.

[27] Lorentz KO, Lemmers SAM, Chrysostomou C, Dirks W, Zaruri RM, Foruzanfar F, et al. First permanent molars with accentuated line patterns: Assessment of childhood health in an urban complex of the fifth millennium before the present. Arch Oral Biol. 2021;123:104969. doi: 10.1016/j.archoralbio.2020.104969 33450640

[28] Austin C, Kumar P, Carter EA, Lee J, Smith TM, Hinde K, et al. Stress exposure histories revealed by biochemical changes along accentuated lines in teeth. Chemosphere. 2023;329:138673. doi: 10.1016/j.chemosphere.2023.138673 37054846 PMC10167648

[29] Nava A, Frayer DW, Bondioli L. Longitudinal analysis of the microscopic dental enamel defects of children in the Imperial Roman community of Portus Romae (necropolis of Isola Sacra, 2nd to 4th century CE, Italy). J Archaeol Sci: Rep. 2019;23:406–15. doi: 10.1016/j.jasrep.2018.11.007

[30] Kierdorf H, Witzel C, Bocaege E, Richter T, Kierdorf U. Assessment of physiological disturbances during pre- and early postnatal development based on microscopic analysis of human deciduous teeth from the Late Epipaleolithic site of Shubayqa 1 (Jordan). Am J Phys Anthropol. 2021;174(1):20–34. doi: 10.1002/ajpa.24156 33017861

[31] Zanolli C, Bondioli L, Manni F, Rossi P, Macchiarelli R. Gestation length, mode of delivery, and neonatal line-thickness variation. Hum Biol. 2011;83(6):695–713. doi: 10.3378/027.083.0603 22276969

[32] Hassett BR, Dean MC, Ring S, Atkinson C, Ness AR, Humphrey L. Effects of maternal, gestational, and perinatal variables on neonatal line width observed in a modern UK birth cohort. Am J Phys Anthropol. 2020;172(2):314–32. doi: 10.1002/ajpa.24042 32155296

[33] Witzel C. Echoes from birth--mutual benefits for physical and forensic anthropology by applying increment counts in enamel of deciduous teeth for aging. Anthropol Anz. 2014;71(1–2):87–103. doi: 10.1127/0003-5548/2014/0386 24818441

[34] Schour I. The Neonatal Line in the Enamel and Dentin of the Human Deciduous Teeth and First Permanent Molar**From the Department of Histology, College of Dentistry, University of Illinois.*Presented in the form of a discussion and demonstration before the Ninth Int. The Journal of the American Dental Association (1922). 1936;23(10):1946–55. doi: 10.14219/jada.archive.1936.0277

[35] Lemmers SAM, Dirks W, Street SE, Ngoubangoye B, Herbert A, Setchell JM. Dental microstructure records life history events: A histological study of mandrills (Mandrillus sphinx) from Gabon. J Hum Evol. 2021;158:103046. doi: 10.1016/j.jhevol.2021.103046 34332420

[36] Dean MC, Elamin F. Parturition lines in modern human wisdom tooth roots: do they exist, can they be characterized and are they useful for retrospective determination of age at first reproduction and/or inter-birth intervals?. Ann Hum Biol. 2014;41(4):358–67. doi: 10.3109/03014460.2014.923047 24932749

[37] Dirks W, Reid DJ, Jolly CJ, Phillips-Conroy JE, Brett FL. Out of the mouths of baboons: stress, life history, and dental development in the Awash National Park hybrid zone, Ethiopia. Am J Phys Anthropol. 2002;118(3):239–52. doi: 10.1002/ajpa.10089 12115280

[38] Schwartz GT, Reid DJ, Dean MC, Zihlman AL. A Faithful Record of Stressful Life Events Recorded in the Dental Developmental Record of a Juvenile Gorilla. Int J Primatol. 2006;27(4):1201–19. doi: 10.1007/s10764-006-9051-2

[39] Austin C, Smith TM, Farahani RMZ, Hinde K, Carter EA, Lee J, et al. Uncovering system-specific stress signatures in primate teeth with multimodal imaging. Sci Rep. 2016;6:18802. doi: 10.1038/srep18802 26727334 PMC4698674

[40] Temple DH. Patterns of systemic stress during the agricultural transition in prehistoric Japan. Am J Phys Anthropol. 2010;142(1):112–24. doi: 10.1002/ajpa.21208 19953616

[41] Birch W, Dean MC. A method of calculating human deciduous crown formation times and of estimating the chronological ages of stressful events occurring during deciduous enamel formation. J Forensic Leg Med. 2014;22 127–44. doi: 10.1016/j.jflm.2013.12.002 24485438

[42] Mountain RV, Zhu Y, Pickett OR, Lussier AA, Goldstein JM, Roffman JL, et al. Association of maternal stress and social support during pregnancy with growth marks in children’s primary tooth enamel. JAMA Netw Open. 2021;4(11):e2129129. doi: 10.1001/jamanetworkopen.2021.29129 34751761 PMC8579236

[43] Kurek M, Borowska B, Lubowiedzka-Gontarek B, Rosset I, Żądzińska E. Disturbances in primary dental enamel in Polish autistic children. Sci Rep. 2020;10(1):12751. doi: 10.1038/s41598-020-69642-3 32728144 PMC7391627

[44] Nava A, Lugli F, Lemmers S, Cerrito P, Mahoney P, Bondioli L, et al. Reading children’s teeth to reconstruct life history and the evolution of human cooperation and cognition: The role of dental enamel microstructure and chemistry. Neurosci Biobehav Rev. 2024;163:105745. doi: 10.1016/j.neubiorev.2024.105745 38825260

[45] Juster R-P, Bizik G, Picard M, Arsenault-Lapierre G, Sindi S, Trepanier L, et al. A transdisciplinary perspective of chronic stress in relation to psychopathology throughout life span development. Dev Psychopathol. 2011;23(3):725–76. doi: 10.1017/S0954579411000289 21756430

[46] Lupien SJ, McEwen BS, Gunnar MR, Heim C. Effects of stress throughout the lifespan on the brain, behaviour and cognition. Nat Rev Neurosci. 2009;10(6):434–45. doi: 10.1038/nrn2639 19401723

[47] McEwen BS. Brain on stress: how the social environment gets under the skin. Proc Natl Acad Sci U S A. 2012;109 (Suppl 2):17180–5. doi: 10.1073/pnas.1121254109 . 23045648 PMC3477378

[48] Cardoso H, Magalhães T. Evidence of neglect from immature human skeletal remains: an auxological approach from bones and teeth. The Juvenile Skeleton In Forensic Abuse Investigations. Springer. 2011:125–50.

[49] Fitzgerald C, Rose J. Reading between the lines: Dental development and subadult age assessment using the microstructural growth markers of teeth. Biological Anthropology Of The Human Skeleton. 2008;237–63.

[50] Smith TM, Reid DJ, Sirianni JE. The accuracy of histological assessments of dental development and age at death. J Anat. 2006;208(1):125–38. doi: 10.1111/j.1469-7580.2006.00500.x . 16420385 PMC2100178

[51] Vacková S, Králík M, Marečková K, Ráčková L, Quade L, Sedláčková L, et al. Human “barcode”: Link between phosphate intensity changes in human enamel and light microscopy record of accentuated lines. Microchem J. 2021;168 106370. doi: 10.1016/j.microc.2021.106370

[52] Thomas R. Enamel defects, well-being and mortality in a medieval Danish village. ProQuest Dissertations Publishing: The Pennsylvania State University; 2003.

[53] Lemmers SAM. Stress, life history and dental development: a histological study of mandrills (Mandrillus sphinx). Durham University, United Kingdom; 2018.

[54] Smith AK, Kilaru V, Klengel T, Mercer KB, Bradley B, Conneely KN, et al. DNA extracted from saliva for methylation studies of psychiatric traits: evidence tissue specificity and relatedness to brain. Am J Med Genet B Neuropsychiatr Genet. 2015;168B(1):36–44. doi: 10.1002/ajmg.b.32278 . 25355443 PMC4610814

[55] Witzel C, Kierdorf U, Schultz M, Kierdorf H. Insights from the inside: histological analysis of abnormal enamel microstructure associated with hypoplastic enamel defects in human teeth. Am J Phys Anthropol. 2008;136(4):400–14. doi: 10.1002/ajpa.20822 . 18350581

[56] Sabel N, Johansson C, Kühnisch J, Robertson A, Steiniger F, Norén JG, et al. Neonatal lines in the enamel of primary teeth—A morphological and scanning electron microscopic investigation. Arch Oral Biol. 2008;53(10):954–63. doi: 10.1016/j.archoralbio.2008.05.003 18589400

[57] Schour I, Massler M. Rate and gradient of growth in human deciduous teeth with special reference to neonatal ring. J Dent Res. 1937;16:349–50.

[58] Guatelli-Steinberg D, Huffman M. Histological features of dental hard tissues and their utility in forensic anthropology. Bone Histology: An Anthropological Perspective. 2017:91–107.

[59] Chandrashekar C, Takahashi M, Miyakawa G. Enamel and Forensic Odontology - Revealing the Identity. J Hard Tissue Biology. 2010;19(1):1–4. doi: 10.2485/jhtb.19.1

[60] Witzel C. Echoes from birth--mutual benefits for physical and forensic anthropology by applying increment counts in enamel of deciduous teeth for aging. Anthropol Anz. 2014;71(1–2):87–103. doi: 10.1127/0003-5548/2014/0386 . 24818441

[61] Rahmat RaA-A N, Nambiar P. Forensic age estimation: Forensic odontology. Clinicopathological Correlation of Oral Diseases: Springer; 2023. pp. 741–50.

[62] Teivens A, Mörnstad H, Norén JG, Gidlund E. Enamel incremental lines as recorders for disease in infancy and their relation to the diagnosis of SIDS. Forensic Sci Int. 1996;81(2–3):175–83. doi: 10.1016/s0379-0738(96)01982-2 . 8837493

[63] Austin C, Kumar P, Carter EA, Lee J, Smith TM, Hinde K, et al. Stress exposure histories revealed by biochemical changes along accentuated lines in teeth. Chemosphere. 2023;329:138673. doi: 10.1016/j.chemosphere.2023.138673 . 37054846 PMC10167648

[64] Kurek M, Borowska B, Lubowiedzka-Gontarek B, Rosset I, Żądzińska E. Disturbances in primary dental enamel in Polish autistic children. Sci Rep. 2020;10(1):12751. doi: 10.1038/s41598-020-69642-3 32728144 PMC7391627

[65] Smith TM, Cook L, Dirks W, Green DR, Austin C. Teeth reveal juvenile diet, health and neurotoxicant exposure retrospectively: What biological rhythms and chemical records tell us. Bioessays. 2021;43(9):e2000298. doi: 10.1002/bies.202000298 . 33721363

[66] Smith TM, Austin C, Green DR, Joannes-Boyau R, Bailey S, Dumitriu D, et al. Wintertime stress, nursing, and lead exposure in Neanderthal children. Sci Adv. 2018;4(10):eaau9483. doi: 10.1126/sciadv.aau9483 . 30402544 PMC6209393

[67] Green DR, Ávila JN, Cote S, Dirks W, Lee D, Poulsen CJ, et al. Fine-scaled climate variation in equatorial Africa revealed by modern and fossil primate teeth. Proc Natl Acad Sci USA. 2022;119(35):e2123366119. doi: 10.1073/pnas.2123366119 . 35994633 PMC9440354

[68] Vaiglova P, Ávila JN, Buckley H, Galipaud JC, Green DR, Halcrow S, et al. Past rainfall patterns in Southeast Asia revealed by microanalysis of δ18O values in human teeth. Journal of Archaeological Science. 2024;162:105922. doi: 10.1016/j.jas.2023.105922

[69] Smith TM, Arora M, Austin C, Nunes Ávila J, Duval M, Lim TT, et al. Oxygen isotopes in orangutan teeth reveal recent and ancient climate variation. Elife. 2024;12 RP90217. doi: 10.7554/eLife.90217 . 38457350 PMC10942278

[70] Kleiner DE, Brunt EM, Van Natta M, Behling C, Contos MJ, Cummings OW, et al. Design and validation of a histological scoring system for nonalcoholic fatty liver disease. Hepatology. 2005;41(6):1313–21. doi: 10.1002/hep.20701 . 15915461

[71] Kottner J, Halfens R, Dassen T. An interrater reliability study of the assessment of pressure ulcer risk using the Braden scale and the classification of pressure ulcers in a home care setting. Int J Nurs Stud. 2009;46(10):1307–12. doi: 10.1016/j.ijnurstu.2009.03.014 . 19406400

[72] Claridge E, Cotton S, Hall P, Moncrieff M. From colour to tissue histology: Physics-based interpretation of images of pigmented skin lesions. Med Image Anal. 2003;7(4):489–502. doi: 10.1016/s1361-8415(03)00033-1 . 14561553

[73] Ibrahim A, Lashen A, Toss M, Mihai R, Rakha E. Assessment of mitotic activity in breast cancer: revisited in the digital pathology era. J Clin Pathol. 2022;75(6):365–72. doi: 10.1136/jclinpath-2021-207742 . 34556501

[74] McHugh ML. Interrater reliability: the kappa statistic. Biochem Med (Zagreb). 2012;22(3):276–82. doi: 10.11613/bm.2012.031 . 23092060 PMC3900052

[75] Gamble J, Ferrel R. Diary of a tooth: harnessing the power of citizen science in bioarchaeology. Annual North American Meeting of Paleopathology Association; Cleveland, Ohio. 2019. doi: DOIoridentifierneeded

[76] Gamble JA, Boldsen JL, Hoppa RD. Stressing out in medieval Denmark: An investigation of dental enamel defects and age at death in two medieval Danish cemeteries. Int J Paleopathol. 2017;17:52–66. doi: 10.1016/j.ijpp.2017.01.001 . 28521912

[77] Rose JC, Armelagos GJ, Lallo JW. Histological enamel indicator of childhood stress in prehistoric skeletal samples. Am J Phys Anthropol. 1978;49(4):511–6. doi: 10.1002/ajpa.1330490411 367176

[78] Żądzińska E, Lorkiewicz W, Kurek M, Borowska-Strugińska B. Accentuated lines in the enamel of primary incisors from skeletal remains: A contribution to the explanation of early childhood mortality in a medieval population from Poland. Am J Phys Anthropol. 2015;157(3):402–10. doi: 10.1002/ajpa.22731 . 25711723

[79] Dirks W, Humphrey LT, Dean MC, Jeffries TE. The relationship of accentuated lines in enamel to weaning stress in juvenile baboons (Papio hamadryas anubis). Folia Primatol (Basel). 2010;81(4):207–23. doi: 10.1159/000321707 . 21124031

[80] Bowman JE. Life history, growth and dental development in young primates: a study using captive rhesus macaques. Cambridge: University of Cambridge. 1991.

[81] Lemmers SAM, Dirks W, Street SE, Ngoubangoye B, Herbert A, Setchell JM. Dental microstructure records life history events: A histological study of mandrills (Mandrillus sphinx) from Gabon. J Hum Evol. 2021;158:103046. doi: 10.1016/j.jhevol.2021.103046 . 34332420

[82] Schwartz GT, Reid DJ, Dean MC, Zihlman AL. A faithful record of stressful life events preserved in the dental developmental record of a juvenile gorilla. Int J Primatol. 2006;27(4):1221–2. doi: 10.1007/s10764-006-9069-5

[83] Gurian KN. What accentuated striae in tooth enamel reveal about developmental stress in two groups of disparate socioeconomic status in Ohio: The Ohio State University. 2021.

[84] Lorentz KO, Lemmers SAM, Chrysostomou C, Dirks W, Zaruri MR, Foruzanfar F, et al. Use of dental microstructure to investigate the role of prenatal and early life physiological stress in age at death. J Archaeol Sci. 2019;104:85–96. doi: 10.1016/j.jas.2019.01.007

[85] McGrath K, El-Zaatari S, Guatelli-Steinberg D, Stanton MA, Reid DJ, Stoinski TS, et al. Quantifying linear enamel hypoplasia in Virunga Mountain gorillas and other great apes. Am J Phys Anthropol. 2018;166(2):337–52. doi: 10.1002/ajpa.23436 . 29460951

[86] McFarlane G, Loch C, Guatelli-Steinberg D, Bayle P, Le Luyer M, Sabel N, et al. Enamel daily secretion rates of deciduous molars from a global sample of children. Arch Oral Biol. 2021;132:105290. doi: 10.1016/j.archoralbio.2021.105290 . 34695672

[87] O’Hara MC, Guatelli-Steinberg D. Differences in enamel defect expression and enamel growth variables in Macaca fascicularis and Trachypithecus cristatus from Sabah, Borneo. Journal of Archaeological Science. 2020;114:105078. doi: 10.1016/j.jas.2020.105078

[88] Guatelli-Steinberg D. Dental stress indicators from micro- to macroscopic. In: Irish JD, Scott GR, editor. A companion to dental anthropology. John Wiley & Sons; 2015, p. 450–64.

[89] Behie AM, Miszkiewicz JJ. Enamel neonatal line thickness in deciduous teeth of Australian children from known maternal health and pregnancy conditions. Early Hum Dev. 2019;137:104821. doi: 10.1016/j.earlhumdev.2019.07.004 . 31330463

[90] Dunn E, Mountain R, Davis K, Shaffer I, Smith A, Roubinov D. Association between measures derived from children’s primary exfoliated teeth and psychopathology symptoms: Results from a community-based study. Front Dent Med. 2022;3:803364. 10.3389/fdmed.2022.803364 PMC13221791 42220472

[91] Caropreso S, Bondioli L, Capannolo D, Cerroni L, MacChiarelli R, Condò SG. Thin sections for hard tissue histology: a new procedure. J Microsc. 2000;199(Pt 3):244–7. doi: 10.1046/j.1365-2818.2000.00731.x . 10971805

[92] Reid DJ, Schwartz GT, Dean C, Chandrasekera MS. A histological reconstruction of dental development in the common chimpanzee, Pan troglodytes. J Hum Evol. 1998;35(4–5):427–48. doi: 10.1006/jhev.1998.0248 . 9774504

[93] Bankhead P, Loughrey MB, Fernández JA, Dombrowski Y, McArt DG, Dunne PD, et al. QuPath: Open source software for digital pathology image analysis. Sci Rep. 2017;7(1):16878. doi: 10.1038/s41598-017-17204-5 . 29203879 PMC5715110

[94] FitzGerald C, Saunders S, Bondioli L, Macchiarelli R. Health of infants in an Imperial Roman skeletal sample: perspective from dental microstructure. Am J Phys Anthropol. 2006;130(2):179–89. doi: 10.1002/ajpa.20275 . 16365859

[95] Peripoli B, Gigante M, Mahoney P, McFarlane G, Coppa A, Lugli F, et al. Exploring prenatal and neonatal life history through dental histology in infants from the Phoenician necropolis of Motya (7th–6th century BCE). J Archaeol Sci: Rep. 2023;49:104024. doi: 10.1016/j.jasrep.2023.104024

[96] Cicchetti DV, Feinstein AR. High agreement but low kappa: II. Resolving the paradoxes. J Clin Epidemiol. 1990;43(6):551–8. doi: 10.1016/0895-4356(90)90159-m . 2189948

[97] Gwet KL. Computing inter-rater reliability and its variance in the presence of high agreement. Br J Math Stat Psychol. 2008;61(Pt 1):29–48. doi: 10.1348/000711006X126600 . 18482474

[98] Vach W, Gerke O. Gwet’s AC1 is not a substitute for Cohen’s kappa - A comparison of basic properties. MethodsX. 2023;10:102212. doi: 10.1016/j.mex.2023.102212 . 37234937 PMC10205778

[99] Tan KS, Yeh Y-C, Adusumilli PS, Travis WD. Quantifying interrater agreement and reliability between thoracic pathologists: Paradoxical behavior of Cohen’s kappa in the presence of a high prevalence of the histopathologic feature in lung cancer. JTO Clin Res Rep. 2023;5(1):100618. doi: 10.1016/j.jtocrr.2023.100618 . 38283651 PMC10820331

[100] Sim J, Wright CC. The kappa statistic in reliability studies: use, interpretation, and sample size requirements. Phys Ther. 2005;85(3):257–68. doi: 10.1093/ptj/85.3.257 . 15733050

[101] Team R. R: A language and environment for statistical computing. R Foundation for Statistical Computing; 2013.

[102] Gamer M. Various coefficients of interrater reliability and agreement. 2010. http://cranr-projectorg/web/packages/irr/irrpdf

[103] Gwet K. Computing chance-corrected agreement coefficients (CAC) version 1.0. 2019.

[104] Bonasia DE, Marmotti A, Massa ADF, Ferro A, Blonna D, Castoldi F, et al. Intra- and inter-observer reliability of ten major histological scoring systems used for the evaluation of in vivo cartilage repair. Knee Surg Sports Traumatol Arthrosc. 2015;23(9):2484–93. doi: 10.1007/s00167-014-2975-8 . 24714975

[105] Robbins P, Pinder S, de Klerk N, Dawkins H, Harvey J, Sterrett G, et al. Histological grading of breast carcinomas: a study of interobserver agreement. Hum Pathol. 1995;26(8):873–9. doi: 10.1016/0046-8177(95)90010-1 . 7635449

[106] Rosenholtz R, Li Y, Nakano L. Measuring visual clutter. J Vis. 2007;7(2):17.1-22. doi: 10.1167/7.2.17 18217832

[107] Whitney D, Levi DM. Visual crowding: A fundamental limit on conscious perception and object recognition. Trends Cogn Sci. 2011;15(4):160–8. doi: 10.1016/j.tics.2011.02.005 . 21420894 PMC3070834

[108] Wolfe JM. Visual Search: How Do We Find What We Are Looking For? Annu Rev Vis Sci. 2020;6:539–62. doi: 10.1146/annurev-vision-091718-015048 . 32320631 PMC13537705

[109] Gillman MW, Blaisdell CJ. Environmental influences on child health outcomes, a research program of the National Institutes of Health. Curr Opin Pediatr. 2018;30(2):260–2. doi: 10.1097/MOP.0000000000000600 . 29356702 PMC6020137

[110] Jernigan TL, Brown SA, Dowling GJ. The adolescent brain cognitive development study. J Res Adolesc. 2018;28(1):154–6. doi: 10.1111/jora.12374 . 29460352 PMC7477916

[111] Martínez de Pinillos M, Pantoja-Pérez A, Fernández-Colón P, Martín-Francés L, García-Campos C, Modesto-Mata M, et al. The Ratón Pérez collection: Modern deciduous human teeth at the Centro Nacional de Investigación sobre la Evolución Humana (Burgos, Spain). Am J Phys Anthropol. 2021;176(3):528–35. doi: 10.1002/ajpa.24371 . 34382686

[112] Le Luyer M, Bayle P. The Tooth Fairy collection (la collection Petite souris), a sample of documented human deciduous teeth at the University of Bordeaux, France. Am J Biol Anthropol. 2022;177(1):175–81. doi: 10.1002/ajpa.24405 . 36787762

[113] Albers J, Pacilé S, Markus MA, Wiart M, Vande Velde G, Tromba G, et al. X-ray-based 3D virtual histology-adding the next dimension to histological analysis. Mol Imaging Biol. 2018;20(5):732–41. doi: 10.1007/s11307-018-1246-3 . 29968183

[114] Antoine D, Hillson S, Dean MC. The developmental clock of dental enamel: a test for the periodicity of prism cross-striations in modern humans and an evaluation of the most likely sources of error in histological studies of this kind. J Anat. 2009;214(1):45–55. doi: 10.1111/j.1469-7580.2008.01010.x . 19166472 PMC2667916

[115] Rudney JD. Dental indicators of growth disturbance in a series of ancient Lower Nubian populations: changes over time. Am J Phys Anthropol. 1983;60(4):463–70. doi: 10.1002/ajpa.1330600408 . 6846517

[116] Cohen S. Artificial intelligence and deep learning in pathology. Elsevier Health Sciences; 2020.

[117] Mire A, Elangovan V, Patil S. Advances in deep learning for medical image analysis. CRC Press; 2022.

[118] Singh NK, Raza K. Progress in deep learning-based dental and maxillofacial image analysis: A systematic review. Expert Syst Appl. 2022;199:116968. doi: 10.1016/j.eswa.2022.116968

[119] Wu Y, Cheng M, Huang S, Pei Z, Zuo Y, Liu J, et al. Recent advances of deep learning for computational histopathology: principles and applications. Cancers (Basel). 2022;14(5):1199. doi: 10.3390/cancers14051199 . 35267505 PMC8909166

[120] Walter SD, Eliasziw M, Donner A. Sample size and optimal designs for reliability studies. Statist Med. 1998;17(1):101–10. doi: 10.1002/(sici)1097-0258(19980115)17:1<101::aid-sim727>3.0.co;2-e 9463853

[121] Wilkinson MD, Dumontier M, Aalbersberg IJJ, Appleton G, Axton M, Baak A, et al. The FAIR Guiding Principles for scientific data management and stewardship. Sci Data. 2016;3:160018. doi: 10.1038/sdata.2016.18 . 26978244 PMC4792175

[122] Birch W, Dean MC. A method of calculating human deciduous crown formation times and of estimating the chronological ages of stressful events occurring during deciduous enamel formation. J Forensic Leg Med. 2014;22:127–44. doi: 10.1016/j.jflm.2013.12.002 . 24485438

[123] Smith TM, Boesch C. Developmental defects in the teeth of three wild chimpanzees from the Taï forest. Am J Phys Anthropol. 2015;157(4):556–70. doi: 10.1002/ajpa.22741 . 25820182

[124] Hodgkins J, Orr CM, Gravel-Miguel C, Riel-Salvatore J, Miller CE, Bondioli L, et al. An infant burial from Arma Veirana in northwestern Italy provides insights into funerary practices and female personhood in early Mesolithic Europe. Sci Rep. 2021;11(1):23735. doi: 10.1038/s41598-021-02804-z . 34907203 PMC8671481

