# Supplementary material for: Inter-rater reliability of stress signatures in exfoliated primary dentition - Improving scientific rigor and reproducibility in histological data collection
Source: PLoS One. 2025 Mar 19;20(3):e0318700. doi: 10.1371/journal.pone.0318700 (PMC11922276; doi:10.1371/journal.pone.0318700)
Supplement: S1 Fig — Panel A = Histological section of an incisor without markings. Panel B = consensus ratings of pair 1, 2, and 3. Red marking = NNL, off-white marking = MAL. (DOCX) [file pone.0318700.s001.docx]

**Supplementary Figure 1: Example of pair ratings where there was high agreement between pairs of raters.***
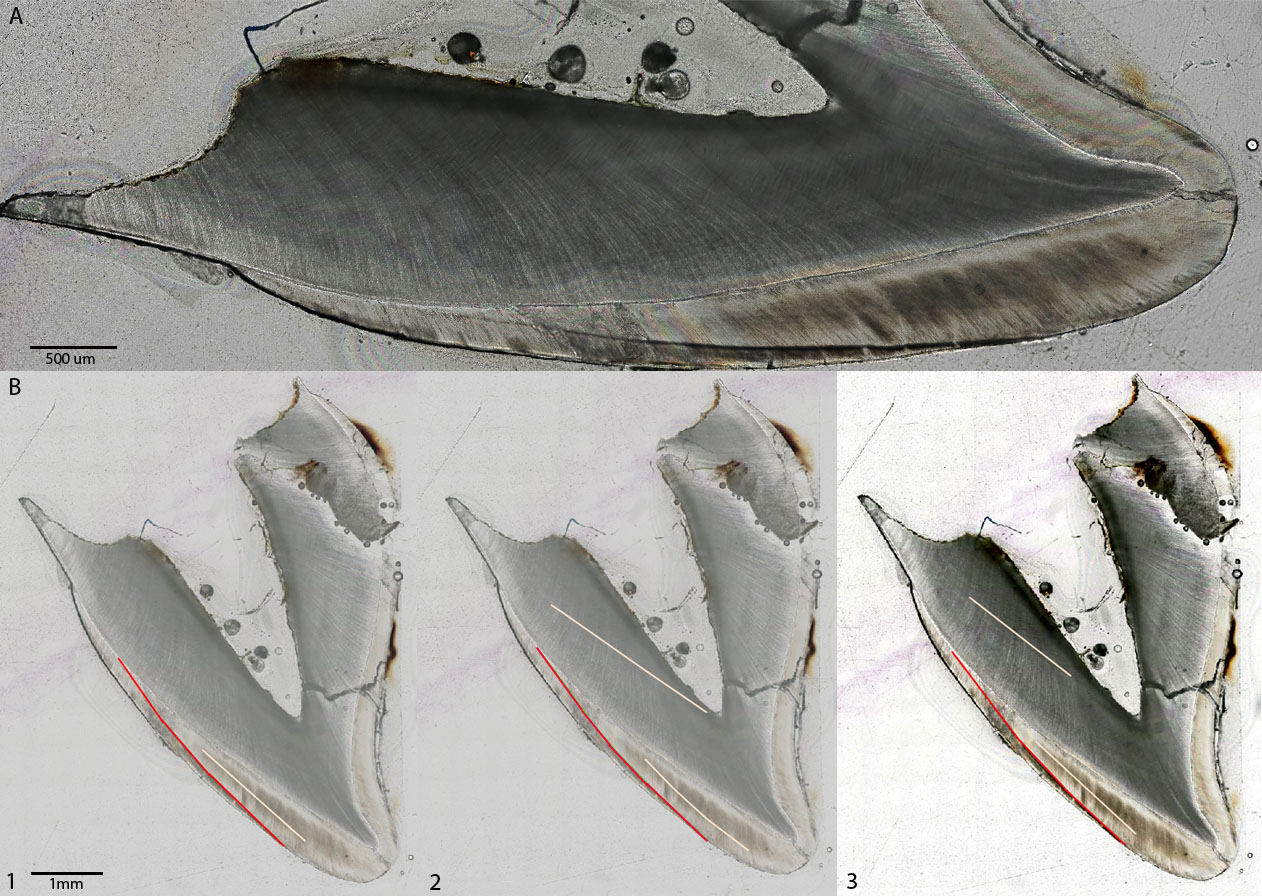


**Panel A= Histological section of an incisor without markings. Panel B= consensus ratings of pair 1, 2, and 3. Red marking= NNL, off-white marking = MAL.*
