# Supplementary material for: Inter-rater reliability of stress signatures in exfoliated primary dentition - Improving scientific rigor and reproducibility in histological data collection
Source: PLoS One. 2025 Mar 19;20(3):e0318700. doi: 10.1371/journal.pone.0318700 (PMC11922276; doi:10.1371/journal.pone.0318700)
Supplement: S2 Fig — Panel A = Histological section of an incisor without markings. Dark brown is the buccal enamel, black areas is dentine. Panel B = consensus ratings of pair 1, 2, and 3. Red = NNL, green = HAL, off-white = MAL. (DOCX) [file pone.0318700.s002.docx]

**Supplementary Figure 2: Example of pair ratings where there was low agreement between pairs of raters.*
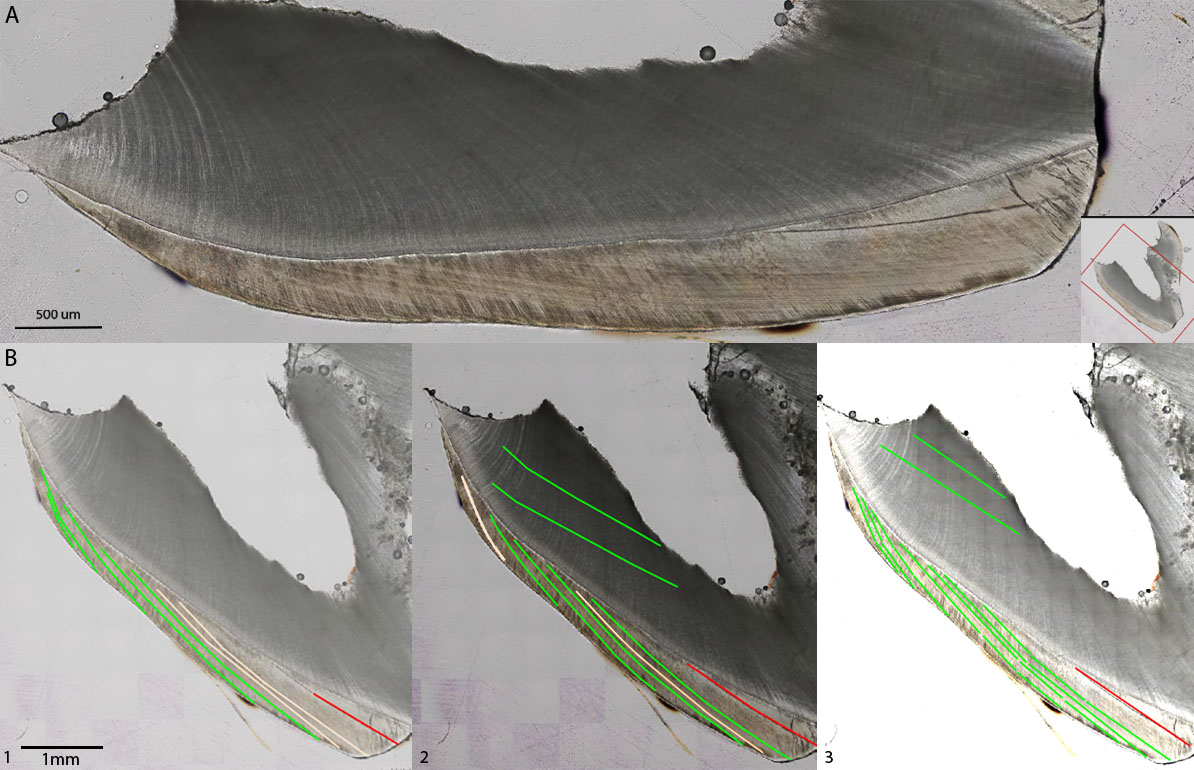
**

**Panel A= Histological section of an incisor without markings. Dark brown is the buccal enamel, black areas is dentine. Panel B= consensus ratings of pair 1, 2, and 3. Red = NNL, green = HAL, off-white = MAL.*
