# Supplementary figures and images for: Inter-rater reliability of stress signatures in exfoliated primary dentition - Improving scientific rigor and reproducibility in histological data collection
Source: PLoS One. 2025 Mar 19;20(3):e0318700. doi: 10.1371/journal.pone.0318700 (PMC11922276; doi:10.1371/journal.pone.0318700)

**Supplementary Figure 3: Results from Approach 1 (GWETs AC1) plotted by reliability coefficient.**


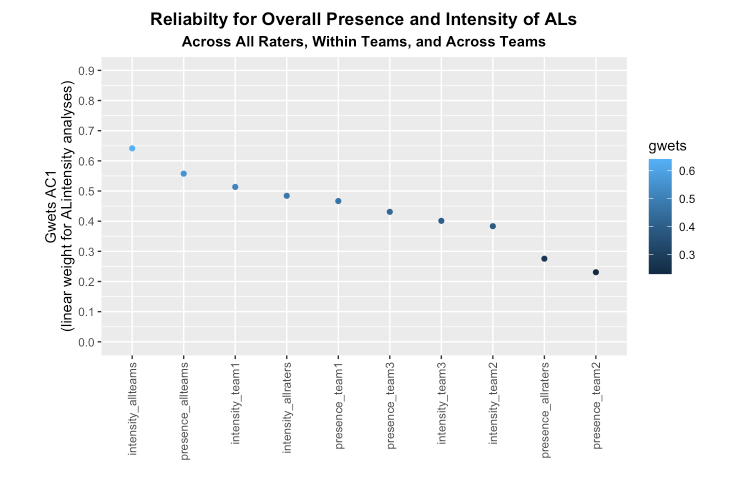

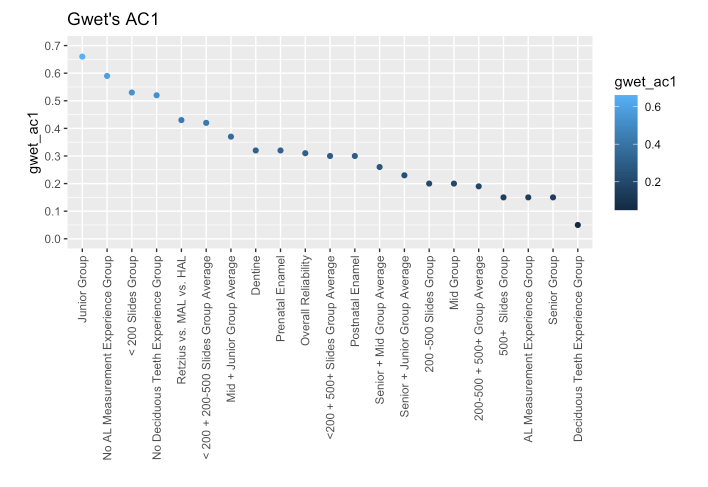

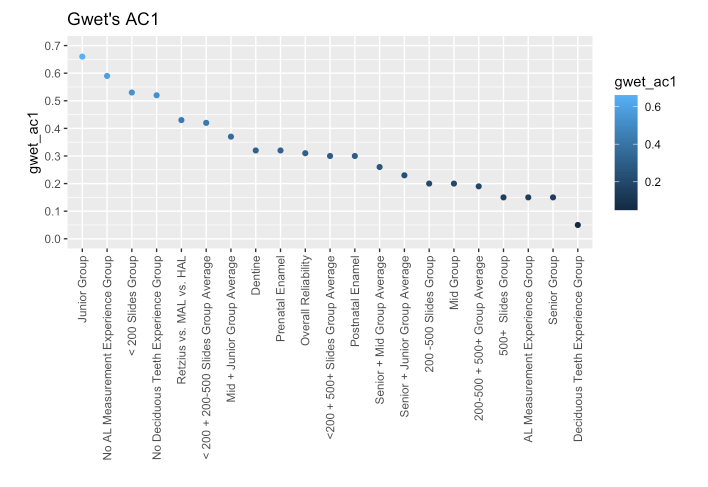

Supplement: S3 Fig — (DOCX) [file pone.0318700.s003.docx]
