# Supplementary material for: Inter-rater reliability of stress signatures in exfoliated primary dentition - Improving scientific rigor and reproducibility in histological data collection
Source: PLoS One. 2025 Mar 19;20(3):e0318700. doi: 10.1371/journal.pone.0318700 (PMC11922276; doi:10.1371/journal.pone.0318700)
Supplement: S4 Fig — (DOCX) [file pone.0318700.s004.docx]

**Supplementary Figure 4: Results from Approach 2 (linear-weighted Gwet’s AC1) plotted by reliability coefficient.**

***
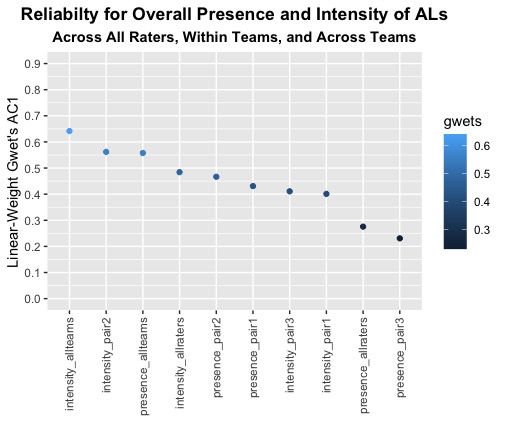
***
