# Supplementary material for: Inter-rater reliability of stress signatures in exfoliated primary dentition - Improving scientific rigor and reproducibility in histological data collection
Source: PLoS One. 2025 Mar 19;20(3):e0318700. doi: 10.1371/journal.pone.0318700 (PMC11922276; doi:10.1371/journal.pone.0318700)
Supplement: S1 Table — (DOCX) [file pone.0318700.s005.docx]

**Supplementary Table 1: Interpretation of Kappa Statistic.***

| Reliability Coefficient | Level of Agreement |
| --- | --- |
| 0 - 0.20 | poor |
| 0.21 - 0.40 | fair |
| 0.41 – 0.60 | moderate |
| 0.61 – 0.80 | good |
| 0.81 –1 | very good |

*Guidelines by Altman (1991), where a κ-value 0.20 is considered poor agreement, 0.21–0.40 fair, 0.41–0.60 moderate, 0.61–0.80 good and 0.81–1 very good.
