# Supplementary material for: Inter-rater reliability of stress signatures in exfoliated primary dentition - Improving scientific rigor and reproducibility in histological data collection
Source: PLoS One. 2025 Mar 19;20(3):e0318700. doi: 10.1371/journal.pone.0318700 (PMC11922276; doi:10.1371/journal.pone.0318700)
Supplement: S2 Table — (DOCX) [file pone.0318700.s006.docx]

**Supplementary Table 2: Overview results from Approach 1 with a further breakdown on type of experience and corresponding inter-rater reliability including kappa coefficients.**

|  | **Line Presence Reliability:** | **Line Presence Reliability** | **Line Intensity Reliability:** | **Line Intensity Reliability** |
| --- | --- | --- | --- | --- |
|  | **Light's Kappa** | **Gwet’s AC1** | **Linear Weighted Light's Kappa** | **Linear Weighted Gwet’s AC1** |
| **All Raters (n=8)** | 0.28 | 0.31 | 0.34 | 0.60 |
| **Seniority** | **---** | **---** | **---** | **---** |
| Senior (n=2) | 0.22 | 0.15 | 0.28 | 0.51 |
| Mid (n=3) | 0.25 | 0.20 | 0.31 | 0.55 |
| Junior (n=3) | 0.35 | 0.66 | 0.42 | 0.80 |
| **AL Measurement Experience** | **---** | **---** | **---** | **---** |
| Experienced (n=4) | 0.18 | 0.15 | 0.28 | 0.51 |
| Unexperienced (n=4) | 0.35 | 0.59 | 0.41 | 0.75 |
| **Deciduous Teeth Experience** | **---** | **---** | **---** | **---** |
| Experienced (n=3) | 0.10 | 0.05 | 0.22 | 0.47 |
| Unexperienced (n=5) | 0.35 | 0.52 | 0.40 | 0.71 |
| **Number of Slides Worked On** | **---** | **---** | **---** | **---** |
| 500+ (n=2) | 0.22 | 0.15 | 0.28 | 0.51 |
| 200 – 500 (n=3) | 0.20 | 0.20 | 0.27 | 0.57 |
| < 200 (n=3) | 0.41 | 0.53 | 0.41 | 0.71 |
